# Peri-Operative Glycemic Dynamics in a Chinese Patient With Type 2 Diabetes Undergoing Laparoscopic Sleeve Gastrectomy

**DOI:** 10.7759/cureus.19029

**Published:** 2021-10-25

**Authors:** Song Wen, Min Gong, Ligang Zhou

**Affiliations:** 1 Endocrinology, Shanghai Pudong Hospital, Shanghai, CHN

**Keywords:** type 2 diabetes, chinese patient, peri-operative glycemic dynamics, laparoscopic sleeve gastrectomy, obesity, continued glucose monitoring system

## Abstract

Obesity and type 2 diabetes (T2D) are prevalent issues in China. Bariatric and metabolic surgery, by reducing the size of the stomach through the removal of a portion of the stomach using laparoscopy (laparoscopic sleeve gastrectomy (LSG)), induces the remarkable remission of T2D inpatients. Plasma glucose (PG) was reported to be at a lower than normal level in Caucasian patients a few weeks after surgery, which is not well-documented in Chinese patients who have a lower body mass index (BMI) compared to Caucasians. Thus, we adopted the use of a continuous glucose monitoring system (CGMS) in a Chinese patient to monitor postoperative glucose levels. We found that the level of PG lowered to the normal range four days after LSG surgery while weight loss was not significantly reduced. It is indicated that the main mechanism of LSG inducing remission of T2DM is the limitation of food intake in addition to the imbalance of a few gastrointestinal hormones such as glucagon-like peptide 1 (GLP-1), Ghrelin. The lower the BMI, the lower the adipose tissue, and the faster the decrease in PG after bariatric and metabolic surgery.

## Introduction

Type 2 diabetes mellitus (T2DM) is the more prevalent type of diabetes, accounting for around 90% of all cases of diabetes, and obesity is the major causative agent of T2DM [1]. According to the survey by the International Diabetes Federation (IDF), 425 million people currently have type 2 diabetes, and there will be 629 million people with diabetes in the World in 2045. The prevalence rate of adult T2DM in 2017 in China is increasing beyond 10.9% [2], with a higher incidence in overweight and obese people [3], which presents a striking issue to the public health undertaking, which is hard to address. The continuous glucose monitoring system can be used to detect interstitial glucose concentration, which approximates plasma glucose and provides 24-hour detailed information on glycemic profiles. Bariatric and metabolic surgery, by reducing the size of the stomach through the resection of a portion of the stomach using laparoscopy (laparoscopic sleeve gastrectomy, LSG), induces the remarkable remission of hyperglycemia and insulin resistance in T2D inpatients. The previous studies suggested that glucose levels during the first 24 hours, especially six hours after metabolic surgeries were significantly higher than baseline, and reduction happens after 24 hours to 72 hours of sleeve gastrectomy (SG). Thus, we adopted using a continuous glucose monitoring system (CGMS) in a Chinese patient to reveal the glucose levels before and after surgery.

## Case presentation

A 47-year-old female presented to the department of endocrinology with complaints of frequent episodes of thirst and fatigue. Her symptoms were relieved by oral drugs (Glibumide 2 mg, QD, and metformin 0.85 g, Bid). She had regularly monitored her blood sugars and been referred to a specialist in the department of endocrinology for 1.5 years. Then, she followed the recommendation to have a subcutaneous injection of Liraglutide (1.2 mg iH, QD) for six months because of recurrent hypoglycemia and overweight. She lost 5 kgs with the GLP-1 analog. However, her bodyweight loss completely regained at the third month of injection. After much counseling, she was given a recommendation for sleeve gastrectomy (SG) for weight loss. For better control of T2D, she requested SG in our department of gastrointestinal surgery.

Due to her body mass index BMI exceeding 30kg/m^2 ^and metabolic syndrome, the surgeons performed SG in this patient. For better observation of her glycemic change, we decided to install a CGMS in this patient. The glycemic level was monitored for seven consecutive days starting two days before surgery using CGMS. The following parameters were evaluated: 24-h mean glucose levels, mean amplitude of glucose excursions, time in range (TIR, 70-80 mg/dl; 3.9-4.4 mmol/L), hyperglycemia (>180 mg/dl; 10 mmol/L), hypoglycemia (<70 mg/dl; 3.9 mmol/L), and total area under the curve (AUC).

**Figure 1:**
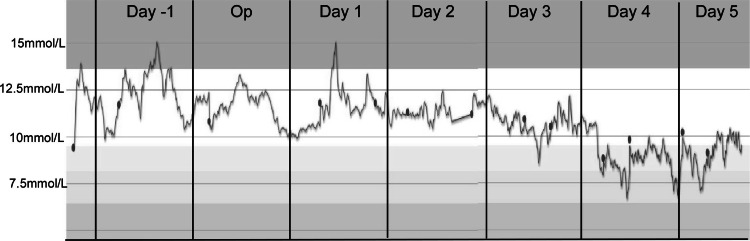
The TIR graph and glucose trend in this patient after sleeve gastrectomy This figure displays the glucose dynamic change before and after the SG sleeve. Day-1: the day before surgery; OP: surgery; Day 1: the first day after surgery; Day 2: the second day after surgery; Day 3: the third day after surgery; Day 4: the fourth day after surgery; Day 5: the fifth day after surgery TIR: time in range; SG: sleeve gastrectomy

We found that the glucose level on the operative day (the day the patient received SG, Op) was lower than that on the preoperative day (Day-1), indicating hyperglycemia (>180 mg/dl; 10 mmol/L) with a peak that slightly exceeded 12.5 mmol/l. However, on Day 1 post-surgery (Day 1), the glucose excursion was substantial and still showed hyperglycemia, with a maximum value in lunchtime of 15 mmol/l. During the next two days, the glucose excursions were stable, and the mean amplitude of glucose excursions was conserved to 10 mmol/l. Dramatically, on the fourth day, a significant remission of hyperglycemia (>10 mmol/l) was seen in this patient (Figure 1).

## Discussion

The continuous glucose monitoring system can be used to detect interstitial glucose concentration, which approximates plasma glucose and provides 24 hours detailed information on glycemic profiles [4]. In this patient, the beneficial effects of SG represented both fasting and postprandial glucose-lowering effect, and this effect was remarkably observed after three days post-surgery.

Data compiled suggest that glucose levels during the first 24 hours, especially six hours after metabolic surgeries were significantly higher than baseline, and reduction happens after 24 hours to 72 hours of SG [5]. Specifically, we find that an exaggerated glucose peak emerged immediately on Day 1 after surgery. We speculated this may be explained by caloric restrictions and increased hormones that drive hyperglycemia in response to stress and absorption fever after surgery. Nonetheless, after three days of surgery, the hyperglycemia acquired remission. Some research indicates that some patients would eventually withdrawal anti-diabetic medications after bariatric surgery (-HbA1c (glycated hemoglobin): -2.1%), and this remission may not be achieved by all individuals [6]. The duration of diabetes may be correlated with the possibility of remission [7]. In this patient, the duration of type 2 diabetes was less than three years, her laboratory checks showed conserved islet function, including insulin secretion, was partially compromised so she was expected to achieve a normal level before operation and withdraw the medication one day after surgery.

In the Asian population, the risk for T2D and cardiovascular disease (CVD) occurs at a lower BMI than in Caucasians [8-9]. This discrepancy may be explained by genetic variation in metabolism or dietary variation, including a high proportion of starch in daily life [10]. Genetic factors likely play a role in obesity. For instance, single nucleotide polymorphisms (SNPs) with a gene such as the melanocortin-4 receptor (MC4R) gene have been determined to lead to class III obesity [11]. Mutation in or near the FTO, PCSK-1, and MC4R genes were found significantly to cause class III obesity with the white and Hispanic groups but less in the Asian and African groups [12]. This can be presented by differences in body composition ethnically, as the Asian group has a higher visceral adipose distribution [13]. In addition, Asian diets are comprised a high percentage of starch, which differs from that of Caucasians. Thus, the cut-point for overweight is lower in the Asian group than that in the Caucasian. A cohort study revealed that a significant mortality risk started at BMI 25 kg/m^2^ in the Asian population, rather than at BMI 30 kg/m^2^ in Caucasians. Therefore, there is evidence that the cut-point for metabolic surgery (Chinese population: BMI>37.5 kg/m^2^ or BMI>32.5 kg/m^2^ with type 2 diabetes; Caucasians: BMI>40 kg/m^2^ or BMI>35 kg/m^2^ with metabolic syndrome) may be different depending upon ethnicity.

## Conclusions

We found that the initiation of remission on hyperglycemia is similar between ethnicities. In this study, we observed it took about three days to attenuate the hyperglycemia. In this study, LSG seems it could effectively improve hyperglycemia in a short duration and maybe could lead to the remission of hyperglycemia or diabetes in this patient. However, it is essential to evaluate if normoglycemia could be maintained in the long term. The long-term evaluation of the glycemia dynamics by CGMS, especially TIR, has more advantages than HbA1c to observe such as glycemic fluctuation; still, it needs further investigation. Therefore, peri-operation CGMS is a useful tool to observe blood glucose fluctuations in patients with type 2 diabetes.
